# Evaluation of Antidiarrheal Activity of Methanolic Extract of *Maranta arundinacea* Linn. Leaves

**DOI:** 10.1155/2015/257057

**Published:** 2015-08-06

**Authors:** Md. Khalilur Rahman, Md. Ashraf Uddin Chowdhury, Mohammed Taufiqual Islam, Md. Anisuzzaman Chowdhury, Muhammad Erfan Uddin, Chandra Datta Sumi

**Affiliations:** ^1^Department of Pharmacology, Dongguk University, Gyeongju 780-714, Republic of Korea; ^2^Department of Systems Immunology, Kangwon National University, Chuncheon 200-701, Republic of Korea; ^3^Department of Pharmacy, Chosun University, Gwangju 61452, Republic of Korea; ^4^Department of Pharmacy, International Islamic University Chittagong, Chittagong 4203, Bangladesh; ^5^Department of Systems Biotechnology, Chung-Ang University, Anseong 456-756, Republic of Korea

## Abstract

Diarrhea is one of the most common causes for thousands of deaths every year. Therefore, identification of new source of antidiarrheal drugs becomes one of the most prominent focuses in modern research. Our aim was to investigate the antidiarrheal and cytotoxic activities of methanolic extract of *Maranta arundinacea* linn. (MEMA) leaves in rats and brine shrimp, respectively. Antidiarrheal effect was evaluated by using castor oil-induced diarrhea, enteropooling, and gastrointestinal motility tests at 200 mg/kg and 400 mg/kg body weight in rats where the cytotoxic activity was justified using brine shrimp lethality bioassay at different concentrations of MEMA. The extract showed considerable antidiarrheal effect by inhibiting 42.67% and 57.75% of diarrheal episode at the doses of 200 and 400 mg/kg, respectively. MEMA also significantly (*p* < 0.01) reduced the castor oil-induced intestinal volume (2.14 ± 0.16 to 1.61 ± 0.12 mL) in enteropooling test as well as intestinal transit (33.00 to 43.36%) in GI motility test, compared to their respective control. These observed effects are comparable to that of standard drug loperamide (5 mg/kg). On the other hand, in brine shrimp lethality test after 24 h, surviving brine shrimp larvae were counted and LD_50_ was assessed. Result showed that MEMA was potent against brine shrimp with LD_50_ value of 420 *µ*g/mL. So the highest dose of 400 *µ*g/mL of MEMA was not toxic to mice. So these results indicate that bioactive compounds are present in methanolic extract of *Maranta arundinacea* leaves including significant antidiarrheal activity and could be accounted for pharmacological effects.

## 1. Introduction

Due to unhygienic livelihood condition, peoples of the third world counties are very prone to several common diseases including diarrhea. According to the World Health Organization (WHO), diarrhea is the second leading reason of death of children less than five years of age [1]. During diarrhea, the normal bowel movement becomes changed, which results in an increase in water content, volume, or frequency of the stools [2]. The common reason for causing diarrhea is gastrointestinal infection by various types of bacteria, virus, and parasites. This infection can be spread out through food, drinking water, and unhygienic environment. Besides other pathological conditions, usually four major mechanisms are responsible for pathophysiology in electrolyte and water transportation, such as increasing of luminal osmolarity and electrolyte secretion, decreasing of electrolyte absorption, and acceleration of intestinal motility ultimately decreasing of transition time [3].

Despite the efforts of international organizations to control this disease, still the incidence of diarrhea is very high [4]. Some antibiotics are used as antidiarrheal drug, but these drugs sometimes show some adverse effects and microorganisms are tend to develop resistance towards them [5]. Therefore the search for safe and more effective agents from plant origin has continued to be an important area of active research. However, plants have long been a very important source of new drugs. Many plant species have been screened for substances with therapeutic activity. For the treatment of diarrhea, medicinal plants are a potential source of antidiarrheal drugs [6]. Moreover, many international organizations including WHO have encouraged studies pertaining to the treatment and prevention of diarrheal diseases using traditional medical practices [7–9]. At present, around 25% of drugs are isolated from plants and there are numerous evidences available about the use of medicinal plants including their pharmacological and biochemical properties [10].

*Maranta arundinacea* Linn. is a tropical and perennial tuberous plant belonging to the family Marantaceae. Locally, this plant is called arrowroot which contains more than 20% of starch in its tubers. It increases digestion and used as nourishing diet for convalescents, mainly in bowel illness. Arrowroot is also popular in traditional medicine for its demulcent properties [11, 12]. However, there are no available medicinal claims about antidiarrheal activity and cytotoxicity of this plant. That is why we are interested in examining the antidiarrheal and cytotoxic activities of methanolic extract of* Maranta arundinacea* L. leaves.

## 2. Materials and Methods

### 2.1. Plant Materials and Extract Preparation

*Maranta arundinacea* L. leaves were collected from Saint Martin Island, Bangladesh, and authenticated by the expert of Bangladesh Forest Research Institute, Chittagong, Bangladesh (Voucher number 5646). The leaves of* Maranta arundinacea* L. were air dried at room temperature and ground into fine powder by pulverization in electric grinder. The powder was successively extracted in methanol (55–60°C) with occasional agitation and then filtered through a cotton plug followed by Whitman Filter Paper number 1. The solvent was evaporated under vacuum at room temperature to yield semisolid extract. Then, the methanolic extract of* Maranta arundinacea* L. (MEMA) leaves was collected in Eppendorf tube and preserved in a refrigerator at 4°C for further use.

### 2.2. Drugs and Chemicals

Castor oil (WELL's Heath Care, Spain), 0.9% sodium chloride solution (normal saline) (Orion Infusions Ltd., Bangladesh), charcoal meal (10% activated charcoal in 5% gum acacia), and loperamide (Square Pharmaceuticals Ltd., Bangladesh) were used for antidiarrheal activity test, and dimethyl sulfoxide (DMSO) (Sigma-Aldrich, USA) and sodium chloride (Sigma) were used for cytotoxic activity test.

### 2.3. Experimental Animals

Long-Evans rats (95–100 g) were collected from International Centre for Diarrheal Disease and Research, Bangladesh (ICDDR, B), which were used as the experimental model for investigation of the antidiarrheal activity. All the animals housed under standard laboratory condition at 25.0 ± 2.0°C and 12 h light: dark cycle, acclimatized for 10 days before experiment. Standard diet and water were provided constantly. All experiments on rat were conducted in accordance with the National Institute of Health Guide for Care and Use of Laboratory Animal (Publication Number 85-23, revised 1985).

### 2.4. Castor Oil-Induced Diarrhea in Rats

Rats of both sexes (95–100 g) were fasted for 18 hours. The selected rats for castor oil-induced diarrheal test were divided into four groups (*n* = 5). Group I was given normal saline (2 mL/kg) orally as control group and Group II received loperamide (5 mg/kg) as standard group. Groups III-IV received MEMA (200 and 400 mg/kg b. wt. i.p., resp.). After 1 h, all groups received castor oil 1 mL each orally. Then they were placed in cages lined with adsorbent papers and observed for 4 h for the presence of characteristic diarrheal droppings. 100% was considered as the total number of feces of control group [13]. The activity was expressed as % inhibition of diarrhea. The percent (%) inhibition of defecation was measured using the following formula:

(1) Percent (%) inhibition of defecation=A−BA×100,

where *A* is mean number of defecation time caused by castor oil and *B* is mean number of defecation time caused by drug or extract.

### 2.5. Castor Oil-Induced Enteropooling

Castor oil-induced enteropooling test helps to determine the prevention of fluid accumulation ability of extract. Here also rats of both sexes (95–100 g) were fasted for 18 hours. The selected rats for this test were divided into four groups (*n* = 5). Group I (controlled group) was given normal saline (2 mL/kg) orally while Group II (standard group) received loperamide (5 mg/kg). The rest of the groups (Groups III-IV) received MEMA (200 and 400 mg/kg b. wt. i.p. resp.). After 1 h, all groups received castor oil, 1 mL orally per animal. Two hours later, all rats were sacrificed and the small intestine from the pylorus to the caecum was isolated. The intestinal contents were collected by milking into a graduated tube and their volume was measured [14].

### 2.6. Gastrointestinal Motility Test

This test was done according to the method of Mascolo et al. and Rahman et al. For this test, selected rats were divided into four groups of five rats in each. At first, 1 mL castor oil was given orally in every rat of each group to produce diarrhea. After 1 h, Group I (control group) received saline (2 mL/kg) orally. Group II received standard drug (loperamide 5 mg/kg b. wt. i.p) and Groups III-IV (the rest of the two groups) received MEMA (200 and 400 mg/kg b. wt. i.p. resp.). After 1 h, all animals received 1 mL of charcoal meal (10% charcoal suspension in 5% gum acacia) orally. One hour after following the charcoal meal administration, all animals were sacrificed and the distance covered by the charcoal meal in the intestine, from the pylorus to the caecum, was measured and expressed as percentage of distance moved [15, 16].

### 2.7. Brine Shrimp Lethality Bioassay

Brine shrimp lethality bioassay was conducted for investigating the cytotoxicity of MEMA. For this test, brine shrimps (*Artemia salina*) were hatched in a round shaped vessel (1 L), filled with sterile artificial seawater (prepared using sea salt of sodium chloride 38 g L^−1^ and adjusted to pH 8.5 using 1 N NaOH) with continuous oxygen supply. After 48 h of hatching, the active nauplii were identified and collected from clear part of the vessel and used for the study. Twenty nauplii were withdrawn through a glass capillary and transferred to each test tube containing 4.5 mL of brine solution. Then 0.5 mL of the extract solution(s) was added to each test tube and kept them in room temperature for 24 h under light. After that time, surviving larvae were counted. This experiment was done along with control (vehicle treated), different concentrations (50–800 *μ*g/mL) [17].

### 2.8. Statistical Analysis

The results are presented as mean  ±  standard error of mean (SEM). The one-way ANOVA test with Dunnett's post hoc test was used to analyze and compare the data using SPSS 11.5 software, while *p* < 0.05–0.001 were considered as statistically significant.

## 3. Results

### 3.1. Castor Oil-Induced Diarrhea

In case of castor oil-induced diarrheal test, the methanol extract of* Maranta arundinacea* L. showed a marked antidiarrheal effect in the rats (Table 1). In both doses, 200 mg/kg and 400 mg/kg, extract produced significant (*p* < 0.01) defecation. The leaves extract doses of 200 mg/kg and 400 mg/kg decrease the total amount of wet feces produced upon administration of castor oil (6.33 ± 0.93 and 5.79 ± 0.52 g) at doses 200 mg/kg and 400 mg/kg compared to the control group (5.00 ± 0.33 g) at the dose of 5 mg/kg.

### 3.2. Castor Oil-Induced Enteropooling

In this test, MEMA at both of the 200 and 400 mg/kg doses produced significant and dose dependent reduction in intestinal weight and volume (Table 2). The leaves extract decreased intestinal volume by 30.33% and 40.16% at doses 200 and 400 mg/kg, respectively. The standard drug loperamide (5 mg/kg) also significantly inhibited (*p* < 0.01) the intestinal fluid accumulation (42.58%).

### 3.3. Gastrointestinal Motility Test

The methanolic extract of* Maranta arundinacea* L. lessened gastrointestinal distance (101 ± 2.82 cm to 57.2 ± 1.41 cm) traveled by the charcoal meal in the rats noticeably compared with the control group. Loperamide (5 mg/kg) produced a marked (46.53%) decrease in the propulsion of charcoal meal through gastrointestinal tract (Table 3).

### 3.4. Brine Shrimp Lethality Bioassay

The cytotoxicity activity of methanolic extract of* M. arundinacea* leaves assayed by the brine shrimp lethality bioassay test. The effect of the extract was dose dependent. In this assay, at 50 *μ*g/mL, MEMA showed the lowest 5% of mortality where, at 800 *μ*g/mL, extract showed the highest 75% of mortality (Table 4). However, at 300 *μ*g/mL concentration, % of mortality was only 20. Overall LD_50_ of MEMA was 420 *μ*g/mL. So, potent bioactive compounds were present in the extract of* M. arundinacea* leaves.

## 4. Discussion

Traditionally, people use plant(s) or plant-derived preparations considering them to be efficacious against diarrheal disorders without any scientific basis [18]. These experimental models were therefore employed to validate antidiarrheal efficacy of methanolic extract of* M. arundinacea* leaves in the current study.

Diarrhea can be described as the abnormally frequent defecation of feces of low consistency which may be due to a disturbance in the transport of water and electrolytes in the intestines. Instead of the multiplicity of etiologies, (i) increased electrolytes secretion (secretory diarrhea), (ii) increased luminal osmolarity (osmotic diarrhea), (iii) deranged intestinal motility causing a decreased transit time, and (iv) decreased electrolytes absorption may be responsible for pathophysiology [19, 20]. Recent study claims that nitric oxide in castor oil is responsible for the diarrheal effect, although it is evidenced that ricinoleic acid produces diarrhea through a hypersecretory response which is the most active component of castor oil [21, 22]. There are several mechanisms proposed to explain the diarrheal effect of castor oil including inhibition of intestinal Na^+^ K^+^ ATPase activity, consequently reducing normal fluid absorption [23, 24], activation of adenylate cyclase or mucosal cAMP-mediated active secretion [25], and stimulation of prostaglandin formation and platelet activating factor [15]. Usually castor oil is metabolized into ricinoleic acid in the gut, which causes irritation and inflammation in the intestinal mucosa, resulting in the release of inflammatory mediators (e.g., prostaglandins and histamine). The released prostaglandins initiate vasodilatation, smooth muscle contraction, and mucus secretion in the small intestines. In experimental animals as well as in human beings, prostaglandins of the E series are considered to be good diarrheagenic agents.

Our study showed that the overall antidiarrheal study reveals the dose dependent activity. In our study, MEMA leaves showed significantly reduced amount of feces in castor oil-induced rat by 44.99% and 55.99% at the doses of 200 and 400 mg/kg, respectively, and % inhibition of diarrhea was 42.67 and 57.57 at 200 and 400 mg/kg, respectively. Moreover, our results directly demonstrate an inhibition of castor oil-induced enteropooling with reduction of the weight and volume of intraluminal contents by 30.33% and 40.16% at 200 and 400 mg/mL, respectively. These results suggest that leaves of* M. arundinacea* contain antidiarrheal components. Also, from these results, it can be predicted that reduction of water and electrolytes secretion into the small intestine may enhance electrolyte absorption from the intestinal lumen consistent with inhibition of hypersecretion [26]. Besides different pathophysiological conditions of diarrhea, hypermotility characterizes diarrhea where the secretory component is not the causative factor [27]. Castor oil produces ricinoleic acid leading to irritation, inflammation of intestinal mucosa, and ultimately diarrhea. At this condition, prostaglandins stimulate gastrointestinal motility and secrete water and electrolytes. It is also well established that loperamide inhibits diarrhea induced by castor oil and charcoal passage test is used to determine the effect of test substance on gut motility [10]. In gastrointestinal motility, MEMA suppressed the propulsive movement or transit of charcoal meal through the gastrointestinal tract which demonstrates that the leaves extract may be able to reduce the frequency of stool in diarrheal conditions.

Previous report on the phytochemical screening on* M. arundinacea* leaves has shown that alkaloids, tannin, cardiac glycosides, steroids, and saponins are absent but phenols and flavonoids are significantly present [28]. It was reported that flavonoids and polyphenols were responsible for the antidiarrheal activity properties [29]. However, previous studies also have shown that flavonoids have ability to inhibit intestinal motility and water and electrolytes secretion [30]. Moreover, in vivo and in vitro tests have also shown that flavonoids are able to inhibit prostaglandin E2 induced intestinal secretion and spasmogens induced contraction and also inhibit release of prostaglandins and autocoids [29]. Thereby, flavonoids as the inhibitors of prostaglandins biosynthesis are considered to delay castor oil-induced diarrhea [31]. Polyphenols also can show antidiarrheal property by interacting and inhibiting cytochrome P450 systems [32]. So, the antidiarrheal activity of the methanolic extract of the leaves of* M. arundinacea* could therefore be due to the presence of flavonoids and phenols.

The brine shrimp lethality test was considered as a convenient probe for primary assessment of toxicity, detection of fungal toxins, heavy metals, and pesticides, and cytotoxicity testing of dental materials. It can also be extrapolated for cell-line toxicity and antitumor activity [17, 32]. MEMA was also assessed for its cytotoxicity using a sensitive in vitro brine shrimp lethality bioassay (Table 4). LD_50_ value of the extract was 420 *μ*g/mL. From this result, it can be well predicted that the* M. arundinacea* extracts do not have considerable cytotoxic activity.

## 5. Conclusion

The findings of the present study provide convincing evidence that methanolic extract of* M. arundinacea* (MEMA) leaves possesses remarkable antidiarrheal activity but has slight cytotoxic effect. Antidiarrheal effect is rapid, long lasting, and statistically significant at both 200 and 400 mg/kg doses. Determination of antidiarrheal effect in other models as well as the effect on gut motility may give a clear idea about the mechanism(s) of antidiarrheal activity. However, further chemical and pharmacological studies are required to isolate the bioactive compounds and elucidate the precise mechanisms responsible for the observed pharmacological activities of this plant.

## Figures and Tables

**Table 1:** Effect of MEMA leaves on castor oil-induced diarrhea in rats.

Group	Treatment	Total number of feces	% Inhibition of defecation	Total number of diarrheal feces	% Inhibition of diarrhea
I	Castor oil + Saline (2 mL/kg p.o)	18.18 ± 1.91^*∗∗*^	—	11.05 ± 1.08^*∗∗*^	—

II	Castor oil + Loperamide (5 mg/kg i.p)	7.76 ± 0.66^*∗∗*^	57.32	5.00 ± 0.33^*∗∗*^	54.75

III	Castor oil + Leaves Extract (200 mg/kg i.p)	10 ± 0.81^*∗∗*^	44.99	6.33 ± 0.93^*∗∗*^	42.67

IV	Castor oil + Leaves Extract (400 mg/kg i.p)	8 ± 1.52^*∗∗*^	55.99	5.79 ± 0.52^*∗∗*^	57.75

Values were expressed as mean ± SEM. (*n* = 5). ^*∗*^ *p* < 0.05, ^*∗∗*^ *p* < 0.01 when compared with control group (ANOVA followed by Dunnett's *t*-test).

**Table 2:** Effect of MEMA leaves on castor oil induced enterpooling in rats.

Group	Treatment	Weight of intestinal content (g)	Volume of intestinal content (mL)	Inhibition (%)
I	Castor oil + Saline (2 mL/kg p.o)	3.22 ± 0.05^*∗∗*^	2.77 ± 0.23^*∗∗*^	—
II	Castor oil + Loperamide (5 mg/kg i.p)	1.84 ± 0.44^*∗∗*^	1.57 ± 0.07^*∗∗*^	42.58
III	Castor oil +Leaves extract (200 mg/kg i.p)	2.46 ± 0.08^*∗*^	2.14 ± 0.016^*∗*^	30.33
IV	Castor oil + Leaves extract (400 mg/kg i.p)	1.92 ± 0.03^*∗∗*^	1.61 ± 0.12^*∗∗*^	40.16

Values were expressed as mean ± SEM. (*n* = 5). ^*∗*^ *p* < 0.05, ^*∗∗*^ *p* < 0.01 when compared with control group (ANOVA followed by Dunnett's *t*-test).

**Table 3:** Effect of MEMA leaves on small intestinal transition in rats.

Group	Treatment	Total length of intestine (cm)	Distance traveled by marker (cm)	Inhibition (%)
I	Castor oil + Saline (2 mL/kg p.o)	107.8 ± 2.36	101 ± 2.82^*∗∗∗*^	—
II	Castor oil + Loperamide (5 mg/kg i.p)	103.36 ± 1.66	44 ± 0.07^*∗∗∗*^	46.53
III	Castor oil + leaves extract (200 mg/kg i.p)	101.03 ± 3.08^*∗∗∗*^	67.6 ± 2.11^*∗∗∗*^	33.00
IV	Castor oil + Leaves extract (400 mg/kg i.p)	93.7 ± 2.61^*∗∗∗*^	57.2 ± 1.41^*∗∗∗*^	43.36

Values were expressed as mean ± SEM. (*n* = 5). ^*∗*^ *p* < 0.05, ^*∗∗*^ *p* < 0.01  ^*∗∗∗*^ *p* < 0.001 when compared with control group (ANOVA followed by Dunnett's *t*-test).

**Table 4:** Cytotoxic effect of MEMA leaves on shrimp nauplii. Cytotoxicity effect of MEMA at various concentrations on the viability of brine shrimp nauplii was examined after 24 hrs incubation.

Concentration (*μ*g/mL)	% of mortality	LD_50_ (*μ*g/mL)
50	5	
100	15	
300	20	420
500	60	
800	75	

All values were the mean of three replicates.
